# Exercise therapy for chronic low back pain: protocol for an individual participant data meta-analysis

**DOI:** 10.1186/2046-4053-1-64

**Published:** 2012-12-21

**Authors:** Jill A Hayden, Jennifer L Cartwright, Richard D Riley, Maurits W vanTulder

**Affiliations:** 1 Department of Community Health & Epidemiology, Dalhousie University, Halifax, Nova Scotia, B3H 1V7, Canada; 2 Nova Scotia Cochrane Resource Centre, Halifax, Nova Scotia, B3H 1V7, Canada; 3 School of Health and Population Sciences, University of Birmingham, Birmingham, B15 2TT, UK; 4 EMGO and Institute for Health and Care Research, VU University, Van der Boechorststraat 7, 1081 BT, Amsterdam, the Netherlands

**Keywords:** Low back pain, Exercise therapy, Meta-analysis, Systematic review

## Abstract

**Background:**

Low back pain (LBP) is one of the leading causes of disability and has a major socioeconomic impact. Despite a large amount of research in the field, there remains uncertainty about the best treatment approach for chronic LBP, and identification of relevant patient subgroups is an important goal. Exercise therapy is a commonly used strategy to treat chronic low back pain and is one of several interventions that evidence suggests is moderately effective.

In parallel with an update of the 2005 Cochrane review, we will undertake an individual participant data (IPD) meta-analysis, which will allow us to standardize analyses across studies and directly derive results, and to examine differential treatment effects across individuals to estimate how patients’ characteristics modify treatment benefit.

**Methods/design:**

We will use standard systematic review methods advocated by the Cochrane Collaboration to identify relevant trials. We will include trials evaluating exercise therapy compared to any or no other interventions in adult non-specific chronic LBP. Our primary outcomes of interest include pain, functional status, and return-to-work/absenteeism. We will assess potential risk of bias for each study meeting selection criteria, using criteria and methods recommended by the Cochrane BRG.

The original individual participant data will be requested from the authors of selected trials having moderate to low risk of bias. We will test original data and compile a master dataset with information about each trial mapped on a pre-specified framework, including reported characteristics of the study sample, exercise therapy characteristics, individual patient characteristics at baseline and all follow-up periods, subgroup and treatment effect modifiers investigated. Our analyses will include descriptive, study-level meta-analysis and meta-regression analyses of the overall treatment effect, and individual-level IPD meta-analyses of treatment effect modification. IPD meta-analyses will be conducted using a one-step approach where the IPD from all studies are modeled simultaneously while accounting for the clustering of participants with studies.

**Discussion:**

We will analyze IPD across a large number of LBP trials. The resulting larger sample size and consistent presentation of data will allow additional analyses to explore patient-level heterogeneity in treatment outcomes and prognosis of chronic LBP.

## Background

Low back pain (LBP) is one of the leading causes of disability and has a major socioeconomic impact [1-5]. The majority of the cost associated with LBP is generated by a small percentage of patients whose condition proceeds to chronicity [6,7]. There is evidence that the prevalence and costs of chronic LBP are rising [8]. Exercise therapy is a commonly used strategy to treat chronic LBP in and is one of several interventions which evidence suggests is moderately effective [9].

In back pain research, identification of relevant patient subgroups is an important goal [10]. Previous interactions with clinical stakeholders identified the lumping together of heterogeneous patients who have non-specific LBP, as a source of frustration in LBP intervention research [11]. There is a presumption that relevant subgroups of individuals with chronic LBP exist, and that our lack of understanding hampers clinical decision-making.

Treatment effect modification occurs when the treatment effect is consistently better for a subgroup of individuals than for the group as a whole. One or more characteristics (treatment effect modifiers) can define treatment-based subgroups (see Kamper *et al*., 2010, for a discussion of treatment-based subgroups) [12]. Promising treatment effect modifiers can come from previous research findings, and clinical or biological rationale. Prognostic factors (characteristics associated with outcome over time) are not necessarily treatment effect modifiers. For LBP there is little conclusive evidence on treatment effect modifiers, although identifying relevant treatment subgroups has been a goal in recent years. LBP has been classified in many ways: on the basis of pathoanatomy, presence/absence of specific signs or symptoms (for example, sciatica), the duration of symptoms (acute, subacute, chronic), work status, diagnostic testing, patient history, or combinations of these. Systems and tools used to classify and subgroup patients with LBP were reviewed by Binkley *et al*. (1993) [13], Fritz *et al*. (2005) [14], and Karayannis (2012) [15]; Kamper *et al*. (2010) [12], discuss research on subgrouping in LBP. These authors report difficulties with most existing subgroup/classification systems, including unclear reliability or validity in clinical practice, non-comprehensive selection of predictor variables, and inclusion of measures or information that are not useful, nor feasibly collected in primary-care practice. Furthermore, most LBP trials are underpowered to detect treatment effect modifiers [16].

Systematic review is a study design that uses transparent and robust methods to search the literature, select appropriate studies, extract relevant data, assess risk of bias, and synthesize and interpret research evidence for a specific question [17]. This is an extremely useful approach to summarize evidence about treatment effectiveness based on a complete body of literature. Meta-analysis, which is the quantitative synthesis of data from primary studies, is valuable to increase the number of patients (statistical power/precision) available to estimate a treatment effect, to better distinguish a clinically important true treatment effect from chance effects, and to identify and investigate sources of between-study heterogeneity in the magnitude of the treatment effect. Traditional meta-analyses that collect published aggregate study data and pool studies to estimate one overall effect have limitations: in particular, they often bring together heterogeneous information which, some argue, limits their relevance to managing individual patients in clinical practice [18]. An alternative approach to evidence synthesis is meta-analysis of individual participant data (IPD), where the raw individual-level data are obtained for each study and used for synthesis. IPD relates to the data recorded for each individual in a study. This is in contrast to aggregate data that relates to information averaged or estimated across all individuals in a study (for example, information on mean treatment effect, mean age, proportion of male participants). Such aggregate data are derived from the IPD itself, so IPD can be considered the original source material. IPD meta-analyses are increasingly achievable [19].

The use of IPD has numerous potential advantages. Aggregate data are often not available, are poorly reported, or are derived and presented differently across studies (for example, odds ratio versus relative risk). They are more likely to be reported (and in greater detail) when statistically significant, amplifying the threat of publication bias. In contrast, IPD allows one to standardize analyses across studies and directly derive the information desired, independent of significance or how it was reported. IPD may also allow a longer follow-up time, more participants, and more outcomes than were considered in the original study publication. This means that IPD meta-analyses are potentially more reliable than aggregate data meta-analyses, and may lead to different conclusions. Perhaps most importantly, an IPD meta-analysis can produce more clinically relevant results, going beyond the grand mean toward individualized medicine and thereby reducing the heterogeneity in study results [20]. For example, subgroups of patients with a common characteristic (for example, female gender) can be identified within IPD, and thus meta-analysis results can be derived specifically for them, with increased power compared to the individual studies themselves. Similarly, IPD allows more powerful and reliable examination of differential treatment effects across individuals [21,22], as one can directly utilize within-trial information to estimate how patients’ characteristics modify treatment benefit [23].

In 2005 our team conducted a review within the framework of the Cochrane Collaboration to investigate the effectiveness of exercise therapy for treating LBP; 61 trials were included [24-26]. We concluded that exercise therapy appears to be effective in slightly decreasing pain and improving function in adults with chronic LBP; however, this earlier work was limited by the availability of only published aggregate data. Since our 2005 Cochrane review, almost 150 new, potentially relevant, randomized controlled trials (RCTs) have been published, warranting an important update. The very large number of recent trials available for this review provides an opportunity for comprehensive and novel syntheses beyond the Cochrane review and traditional meta-analyses. For exercise therapy, one small study is available that attempted to identify individual characteristics for patients likely to respond to stabilization exercises [27]. This highly cited study included 54 subjects and developed a clinical predictive rule. However, these results are preliminary. Chou *et al*. (2010) note, More research on methods for selecting optimal therapy that are practical for use by primary care clinicians is urgently needed. [28]. In this study we will investigate individual characteristics that may modify treatment outcomes in exercise therapy.

## Study objectives

Our primary objective in this project is to assess treatment effect and effect modification of exercise therapy for reducing pain and disability in adults with chronic LBP. We aim to identify subgroups of patients with LBP who are more likely to benefit from specific approaches of exercise therapy.

## Methods/design

### Identifying studies for systematic review

We will use standard systematic review methods advocated by the Cochrane Back Review Group (BRG) to identify relevant trials [29]. Complete descriptions of systematic review methods for the related Cochrane review are reported elsewhere [30]. The search strategy will include a computerized search of electronic databases since the last Cochrane update (2004 to current): MEDLINE, EMBASE, PsychINFO, CINAHL, PEDro, SportDiscus, and the Cochrane Central Register of Controlled Trials. We will conduct citation searches of previous review publications [24-26] and screen cited references of other exercise therapy systematic reviews. We will contact content experts for additional trials. Hand searches of key musculoskeletal journals are captured in the Cochrane Central Register searches.

We will not restrict the searches or inclusion criteria to any specific languages. A standard protocol will be followed for study selection and data abstraction [29]. This includes two reviewers’ independent assessments of study eligibility, data extraction, trial quality and clinical relevance. Consensus and, if needed, a third reviewer will be used to resolve disagreements.

We will identify and include RCTs evaluating exercise therapy compared to any or no other interventions in adult (> 18 years of age) non-specific (alone or with leg pain) chronic (> 12 weeks duration) LBP. Trials with mixed subacute (> 6 weeks duration) and chronic LBP populations will also be eligible for the IPD meta-analysis as it will be possible to extract information on chronic participants. We will exclude studies that involve individuals with LBP caused by specific pathologies (for example, fracture, rheumatoid arthritis, infection, neoplasm, or metastasis) or conditions (for example, pregnancy). Exercise therapy is defined as a supervised exercise program or formal home exercise regimen, ranging from programs aimed at general physical fitness or aerobic exercise, to those aimed at muscle strengthening or stretching, and graded activity programs.

Our primary outcomes of interest include pain, functional status, and return-to-work/absenteeism. Secondary outcomes of interest include global improvement/perceived recovery, health-related quality of life, satisfaction with treatment, reduction in frequency of analgesic use, psychological measures (for example, self-efficacy, fear, catastrophizing, mood) and adverse events. We will extract outcome assessment data for all time periods and group them for the purposes of disseminating the analyses: short-term (post-treatment assessment; 6 to 12 weeks after randomization), medium-term (closest to six months), and long-term follow-up (12 months or more) measured from randomization. Pain and physical function outcomes will be measured as continuous variables and each study’s results will be placed on a common 0 to 100 scale to facilitate comparison and interpretability of the syntheses; return to work/absenteeism outcomes will be measured as time-to-event, if possible, or as dichotomous data.

### Data collection and management

#### Collection of aggregate data

We will extract relevant study data, including population, intervention(s), comparison(s) and outcome information (measure and timing) for each study, and deposit these onto pre-tested standardized electronic (Microsoft Access) forms. One author will extract data and a second author will check data for accuracy. In prior work we identified important intervention characteristics with the assistance of clinical experts. Interventions will be characterized by the exercise program design (individually designed, partially individually designed, or standard), delivery type (home exercises, supervised home, group supervision, or individual supervision), dose/intensity, inclusion of additional interventions, and the type(s) of exercises (for example, muscle strengthening, stretching, coordination, mobilizing, flexibility) (described in detail in Hayden *et al*., 2005) [26]. We will contact primary study authors for additional information when necessary.

We will assess potential risk of bias for each study meeting selection criteria. Risk of bias will be assessed by two independent reviewers, with consensus, using criteria recommended by the Cochrane BRG [29]. We will assess potential bias related to random sequence generation, allocation of treatment concealment, blinding of participants and personnel, blinding of outcome assessment, incomplete outcome data, selective outcome reporting, and other sources of potential bias (similar groups and co-interventions, compliance, timing). For the IPD meta-analysis we will identify the subgroup of selected trials that are rated as moderate to low risk of bias defined as at least six of eleven items rated as low risk of bias with no fatal flaws. We will judge the likelihood of fatal flaws following Cochrane Handbook recommendations, including: 1) a drop-out rate greater than 50% at the follow-up measurement period of interest; 2) clinically relevant baseline differences for one or more primary outcomes indicating unsuccessful randomization; or 3) unacceptable adherence to the exercise program (defined as less than 50% adherence in supervised programs).

### Collection of individual patient data

#### Identifying IPD studies

The original IPD will be requested from the authors of selected studies with moderate to low risk of bias. We will identify contact information for study authors from PubMed or from the Internet and will email authors listed as contact authors to tell them about our IPD meta-analysis, and to ask if they are willing to share their trial data. If there is no response from the contact author, another investigator from the study will be contacted.

We have previously been in contact with authors of included trials in this Cochrane review (for the purpose of obtaining further information and clarification, as well as to explore request of IPD from a small subset of studies). In most cases we have received prompt assistance from the authors, particularly from recently published trials. We requested IPD from 12 high quality trials included in the 2005 Cochrane Review update. We received immediate responses from eleven of the twelve authors and received datasets from four of these. Authors who did not provide data reported no longer having access to the data (many trials were > 5 years since publication). Based on our prior experience, we expect to identify approximately 30 to 40 higher quality trials and anticipate having access to 20 to 30 datasets.

We will make four successful delivery attempts to contact study authors (that is, two attempts each to the listed contact author, and if needed, another study author). Study authors not responding or unwilling to contribute their study data will be sent a final note inquiring why they are unable to participate.

#### Collecting data

We will extract information about each eligible and included IPD study, including the following additional study-level information: reported characteristics of the study sample, variables collected at baseline and follow-up periods, and subgroup and treatment effect modifiers investigated and presented in the report.

We will contact participating study authors to provide additional information about the study and how to send us their IPD. Methods for receiving raw data from investigators may vary depending on the security concerns of their individual institutions; however, data may be obtained by mail (via password-protected memory key and couriered to Dalhousie University, Halifax, Canada), by e-mail, or by a secure transfer system similar to the Dalhousie File Exchange. After data have been received, they will be stored on a secure institutional server. We will accept databases in all formats in order to minimize the amount of work for primary study authors; however, ideally the format will be a two dimensional spreadsheet format with one subject per row, and variables listed in columns.

Each raw dataset will be saved in its original format and then converted to a common format. We will use these common datasets to rename and label the variables for each included study in a consistent manner. We will use a pre-specified preliminary framework for mapping and classifying sufficiently similar variables (Table 1). If in doubt, we will contact primary study authors for clarification and/or discuss within the collaborative group.

**Table 1:** **Preliminary list of potential baseline variables and constructs (based on Hayden, 2007 Appendix) **[31]

**Baseline variable construct**	**Description/examples of potential measures**
**Individual subject characteristics**	
Age	Current age
Sex	Male, female
Body mass index (BMI) or height and weight	BMI or measured height and individual weight
**Lifestyle factors**	
Recreational participation	Participation in sports activities or hobbies
Physical fitness	Physical fitness level
Smoking	Smoking habits
Alcohol consumption	Amount/frequency of drinking, how often drunk
Coffee consumption	Coffee consumed per day
**Sociodemographic characteristics**	
Socio-economic status	Education status, income
Work characteristics	Employed, occupation
Social support	Marital status
Overall health	
General health status	Perceived or self-rated health, general health question or Short Form-12, perceived energy level
Comorbidities	Presence and type of co-morbidities (for example, musculoskeletal pain other than LBP, respiratory or stomach problems, migraines, multiple pain sites)
Previous injuries	Previous sick leave (any cause or other than LBP), or accident
**Receipt of compensation or litigation**	
Worker’s compensation; time off work; sick leave; benefits; pension	Receipt of worker’s compensation, duration of time off work, sick leave due to LBP, benefits paid, pension application applied for or intending to apply for
Attribution for LBP	Litigation, culpability for injury, job that caused pain, description of injury event
**Previous low back pain (before the current episode)**	
Previous history of LBP	Report of prior LBP
	Previous LBP treatment received (for example, injections, inpatient treatment, surgery)
	Prior claim for LBP, LBP sick leave, worker’s compensation injury
	Hospitalization due to LBP, previous lumbar spine radiography
**Characteristics of the current LBP episode**	
Pain onset	Onset of injury (for example, sudden or gradual), injury event or none
Duration of complaint	LBP duration, time between injury and filing claim
**Baseline LBP symptoms**	
Pain severity	Injury severity rating, presence of disabling pain, visual analogue scale, presence of pain at night, NRS; McGill scale
Functional limitations	Disability score (for example, RMDQ, Oswestry), assessment of functioning in leisure time
Change in symptoms	Symptoms getting worse or better
**Baseline physical examination findings**	
Range of motion (ROM)	Change in ROM
Pain pattern (centralization)	Pain pattern/directional preference according to McKenzie method
Other examination findings	Summed physical exam, SLR, muscle palpation, gait, posture, walk test, ‘catch’, Waddell symptoms
**Baseline neurological findings**	
Localization; nerve root; radiculopathy; pain on coughing	Localization of pain to low back only, presence of nerve root signs/symptoms, clinical impression of radiculopathy with SLR, pain on coughing
**Subject psychological status**	
Depression	Rating scale of ‘feeling blue’, Beck Depression Inventory, GHQ depression scale, Zung Self-Rating Depression Scale
Other psychological diagnoses	For example, hysteria, somatic symptoms, hypochondriasis
Other psychological characteristics and behaviors	For example, self-esteem, pain behaviors, denial, state of anxiety, sleep problems, locus of control, fear avoidance beliefs, personality measures, active/passive coping mechanisms
**LBP diagnosis received**	
Diagnostic categories	Specific diagnostic categories (for example, sprain/strain, disc herniation)
Understanding of symptoms	Patient’s understanding and accuracy of their symptoms
**Subject expectations of recovery**	
Subjective work ability	Subjective work capacity in relation to complaint
Perceived ability to do job	Perceptions regarding ability to do job, expectations with injury
Psychosocial capacity to RTW	Psychosocial capacity to RTW, Worker Role Interview
Intent/expectations to RTW	Intent/expectations to return to job
Other baseline/LBP episode characteristics	

NRS: numeric rating scale.

RMDQ: Roland Morris Disability Questionnaire.

SLR: straight leg raise.

GHQ: general health questionnaire.

RTW: return to work.

#### Checking data

We will evaluate data from each study and compare these to available publication(s). We will check each dataset for the range of included variables to make sure all values are reasonable. We will assess missing observations for each variable and check against the original publication. We will attempt to replicate results reported in the original publication, including baseline characteristics and outcome data at each available follow-up period, by reproducing the statistical methods as reported by the study authors. We will discuss and clarify any discrepancies or missing information between our results and those presented in each original publication with the original study authors. If we are unable to reproduce published trial findings or explain discrepancies, these trials will not be included in our IPD synthesis.

Once data checks are complete and satisfactory, individual study datasets will be combined to form a new master dataset with a variable added to indicate the original study.

#### Preparing data for analyses

The complete master database will be maintained for future collaborative initiatives as long as study authors are in agreement. As described below, we intend that the collaborative group will grow and continue to collaborate with future pre-specified analyses. For the current analysis a subset of the master database will be identified, including studies that collected data on pre-specified baseline factors. This will allow multivariable regression analyses that are theory-driven and evidence-informed with an aim to replicate and extend knowledge about treatment effect modifiers for chronic LBP. We will collect data from variables in the following domains: subject characteristics, lifestyle factors, sociodemographic characteristics, overall health, receipt of compensation/litigation, previous LBP, characteristics of the current episode, physical examination findings, psychological status, expectations (see Table 1). Whenever possible, we will maintain data for continuous measurement of variables. For all variables, we will preferentially select measures that are most reliable and have minimal measurement error. If data on a variable of interest are not available in the dataset, we will attempt to extract this information based on other data in the set.

We will assess subject-level missing data on variables and outcomes. Individual subjects with missing outcome data within each trial will generally be excluded from that specific analysis, though we will check that such exclusion does not impact upon baseline balance. Missing baseline variable data will be handled using multiple imputation techniques, under a missing-at-random assumption, so as to avoid excluding patients from the analysis and to ensure baseline balance between treatment groups is maintained [32,33].

### Synthesis strategy

#### Descriptive analyses

We will describe study-level and patient-level characteristics of included studies. We will compare study-level characteristics and aggregate data from studies participating in the IPD analysis with those from studies that are eligible but do not supply data to the collaborative; we will examine if the IPD studies available are a representative (unbiased) sample of the full set of existing studies, as recommended by Ahmed *et al*. (2012) [34].

#### Study-level meta-analysis and meta-regression analyses of the overall treatment effect

Meta-analysis and meta-regression analyses based on aggregate data presented in the publications of primary studies will be conducted as part of the associated ongoing Cochrane review; we will synthesize the trial data on the effect of exercise treatment, and assess the impact of study-level variables including exercise intervention characteristics. These methods are described elsewhere [30], and include (separately for each outcome and each intervention) a random-effects meta-analysis to estimate the pooled (average) treatment effect and its 95% CI [35]; the amount of between-study heterogeneity (quantified by *I*^2^ and tau-squared) [36]; and a 95% prediction interval for the potential treatment effect in a single clinical setting [37].

Additional analyses (again, separately for each outcome and each intervention) will repeat the meta-analyses using the primary trial IPD available. These IPD meta-analyses will be conducted using a one-step approach, where the IPD from all studies are modeled simultaneously while accounting for the clustering of participants within studies. The model used will relate to the type of outcome being synthesized, but will include a linear analysis of covariance model (for continuous outcomes adjusting for baseline value), a logistic regression model (for binary outcomes), and a Cox regression or related survival model (for time-to-event outcomes). The pooled treatment effect of exercise therapy will be estimated according to a mean difference (for continuous outcomes), an odds ratio (for binary outcomes) and a hazard ratio (for time-to-event outcomes) and their 95% CIs, based on the intention-to-treat (ITT) principle in the database that includes IPD from all trials, clustered by trial. These analyses will allow us to compare results using the IPD studies with results using aggregate data from the full set of studies, to ascertain if and how the IPD analysis alone differs to an aggregate data meta-analysis of all studies in terms of the treatment effect obtained.

Where possible, for each analysis we will compare the effect of each exercise therapy considering the duration of the exercise program and any outcome follow-up (time course). We will recognize that this is a study-level comparison, and thus subject to potential study-level confounding.

#### Individual-level IPD meta-analyses of treatment effect modification

In addition to the analyses above, the IPD studies will be used to examine treatment effect modification at patient level, where individual patient characteristics are associated with changes in the treatment response. Candidate predictors of treatment response may be identified by considering biological (including behavioral and sociocultural) or other mechanisms for modification of treatment response (biological reasoning and by understanding the mechanism by which response is modified), and from existing prognostic research (treatment effect modification studies [38] and prognostic factor research [39]).

We will consider the (limited) LBP research evidence on treatment effect modifiers, and prognostic factors with causal or mechanistically relevant effects. Candidate predictors of treatment response that we will consider in our primary analyses include age, duration of symptoms, severity of symptoms/bothersomeness, radicular signs/symptoms, leg symptoms, directional preference, fear avoidance beliefs, depression, social support, general health, acceptance of treatment, and popular clinical prediction models, for example, STarT Back (Hill, 2010) [40], or Delitto’s treatment-based classification (Brennan, 2006) [41].

For each analysis of the overall treatment effect described above, we will identify studies that have additional data for baseline variables that are potential (candidate) predictors of treatment response for one or more of the outcomes of interest. For each of these candidate predictors of treatment response we will present treatment effects for subgroups defined by the predictor, and test for an interaction between each predictor and the effect of treatment on pain and disability outcomes. We will extend the one-step IPD meta-analysis framework described above to include multiple variables and interaction terms between treatment and each variable; the analysis will again account for the clustering of patients within studies, and carefully separate out within-study interaction terms (patient-level) and between-study interaction terms (study-level) to avoid ecological bias [42,43]. We will consider a variable as a clinically important effect modifier if the within-study interaction coefficient is statistically significant at *P* < 0.05 and if the subgroup treatment effects differ by either 10% or more, or another magnitude deemed clinically important by experts. On the basis of current literature on minimal clinically important differences, we will consider an average 20-point (/100) improvement in pain [44] and 10-point (/100) improvement in functioning outcomes [45] to be clinically important. We will consider clinically important individual patient response as any improvement in score ≥ 30% of its baseline value, with a minimum value of 20-point (/100) improvement in pain and 10-point (/100) improvement in functioning [46,47].

#### Sensitivity analysis

The robustness of conclusions to the exclusion of studies without a low risk of bias will also be examined in a sensitivity analysis.

#### Investigation of small study effects

For the overall treatment effect investigations, any meta-analysis containing 10 or more studies will be examined for small study effects, that is, the tendency for smaller studies to provide more significant and positive findings than larger studies. For this purpose contour enhanced funnel plots [48] and tests for funnel plot asymmetry will be utilized, according to recent recommendations [49].

#### Statistical software

Descriptive and aggregate level data analyses will be conducted using Stata (StataCorp LP, Texas, USA), and RevMan 5 (The Nordic Cochrane Centre, Copenhagen, Denmark) software. The one-stage IPD meta-analysis models will primarily be conducted using Stata or SAS. However, if model complexity warrants, a Bayesian Markov Chain Monte Carlo method will also be considered and fitted using the Bayesian software, WinBUGS [50,51]. All IPD analyses conducted will be based on the checked and updated IPD from all eligible available trials. Study data will not be used for any other purpose without the permission of collaborators.

### IPD MA group collaboration

The local project team is responsible for the project’s management decisions and daily management of the collaboration. The project team developed the initial protocol and will organize interactions with the collaborative group. The project team will act as a liaison between members of the collaborative group.

The collaborative group, the Chronic Low Back Pain IPD Meta-Analysis Group (LBP-IPD Group), will be composed of a representative from each of the included trials. We will invite new collaborators as new eligible studies are completed.

Members of the LBP-IPD Group will be given opportunities to participate in decision-making regarding the study design and analyses. A primary publication of the results of this review will be prepared by the project team, and circulated to the collaborative group for critical comment. We will follow recommendations for authorship in IPD analyses of Stewart & Tierney (2002) [52], where core project team members and the collaborative group (the LBP-IPD Group) are listed as authors. Participating members of the collaborative group will be named in acknowledgement.

Members of the LBP-IPD Group will have opportunities to network and participate in future collaborative projects. We intend to pursue other important questions suitable for analysis with the IPD dataset, some led by other members of the collaborative. Once the collaborative group and initial dataset are established we will develop mechanisms for communication and input on methodological issues.

### Ethics

The protocol for this IPD meta-analysis study has been approved by the Dalhousie University Research Ethics Board (REB). We will be synthesizing anonymous data from previous clinical trials where consent will already have been obtained by the original investigators. The REB waived the requirement for obtaining additional informed consent from participants, as the risks to participants are minimal (data have already been collected and published), obtaining consent is practicably impossible (our review will draw from studies that have been conducted in different countries, with different populations, some years past), no therapeutic intervention is involved in the research (the original clinical studies will have administered various treatments or interventions to their participants, but we are not doing so), and the waiver does not violate the rights or well-being of the participants (all participants have already completed their treatment and will have signed consent forms at the time of the original studies). The principal author on each study contributing data will be asked to sign a consent form giving the project team permission to use their data and specifying any restrictions on data usage/storage they may wish to impose.

## Discussion

In this project we will establish a collaborative group and explore and analyze methods and results across a large number of RCTs in the field of LBP. This and related projects [30] will provide a unique opportunity to investigate research methods and gaps in the literature, including comparing results of meta-analysis using standard aggregate-level approaches, multi-treatment meta-regression, and IPD. This project, as well as future collaborative projects, will also help improve quality, design and reporting of LBP trials with respect to collection of information on prognostic factors relevant to the identification of treatment subgroups (similar to the very successful IMPACT project) [53].

The advanced methods that we propose in this project use raw data from RCTs of exercise therapy to explore patient characteristics and identify subgroups based on their likely response to treatments. This approach is not possible using meta-analysis of published aggregate data, as subgroup effects are prone to bias and selective reporting (they are not usually the primary objective of the original trial). Furthermore, a meta-analysis of aggregate data has low power to detect true effect modifiers and is subject to study-level confounding and ecological bias [19,54]. These problems are avoided by using individual-level data.

There are several advantages of IPD meta-analysis that we will exploit in this study: first, the availability of data (increased sample size, more follow-up data); second, consistent presentation of data (direct derivation of information independent of reporting, and standardization of analyses across studies will mean more usable data for meta-analyses), and third, additional analyses to explore heterogeneity (more extensive use of available data to explore study-level and patient-level factors in meta-analyses, and assessment of the variation in summary effect within patient subgroups to allow better understanding of the effects of treatments). This should lead to more reliable and clinically informative meta-analysis results.

## Abbreviations

Cochrane BRG: Cochrane Back Review Group; IPD: individual participant data; LBP: low back pain; LBP-IPD Group: Chronic Low Back Pain IPD Meta-analysis Group; NSHRF: Nova Scotia Health Research Foundation; RCT: randomized controlled trial; REB: Research Ethics Board.

## Competing interests

MWvT and members of the Chronic LBP IPD Meta-analysis Group are investigators of the individual trials included in the IPD dataset. JAH’s Professorship is funded by the Canadian Chiropractic Research Foundation and Dalhousie University.

## Authors’ contributions

JAH conceived the protocol. JAH and JLC developed and drafted the initial protocol with input from RDR and MWvT. All members of the Chronic LBP IPD Meta-analysis Group were sent draft versions and invited to comment and contribute changes. All authors read and approved the final protocol manuscript.

## Authors’ information

Chronic Low Back Pain IPD Meta-Analysis Group (as of October 2012) (in alphabetical order): Tom Bendix (Denmark), Melinda Cairns (UK), Francesca Cecchi (Italy), Leonardo Costa (Brazil/Australia), Ninna Dufour (Denmark), Manuela Ferreira (Australia), Nadine Foster (UK), Helen Frost (UK), Ram Gudavalli (USA), Jan Hartvigsen (Denmark), Pieter Helmhout (the Netherlands), Jan Kool (Switzerland), George Koumantakis (Greece/UK), Francisco Kovacs (Spain), Tiina Kuukkanen (Finland), Audrey Long (Canada), Luciana Machado (Brazil), Chris Maher (Australia), Wolf Mehling (USA), Eva Rasmussen-Barr (Sweden), Cormac Ryan (UK), Karen Sherman (USA), Tuulikki Sjögren (Finland), Rob Smeets (the Netherlands), Bart Staal (the Netherlands), Monica Unsgaard-Tøndel (Norway), Miriam Vollenbroek (the Netherlands).
